# Details of a prospective protocol for a collaborative meta-analysis of individual participant data from all randomized trials of intravenous rt-PA vs. control: statistical analysis plan for the Stroke Thrombolysis Trialists' Collaborative meta-analysis

**DOI:** 10.1111/ijs.12040

**Published:** 2013-05-03

**Authors:** The Stroke Thrombolysis Trialists' Collaborative Group

**Keywords:** acute stroke therapy, clinical trial, rt-PA, stroke, thrombolysis, treatment

## Abstract

**Rationale:**

Thrombolysis with intravenous alteplase is both effective and safe when administered to particular types of patient within 4·5 hours of having an ischemic stroke. However, the extent to which effects might vary in different types of patient is uncertain.

**Aims and Design:**

We describe the protocol for an updated individual patient data meta-analysis of trials of intravenous alteplase, including results from the recently reported third International Stroke Trial, in which a wide range of patients enrolled up to six-hours after stroke onset were randomized to alteplase vs. control.

**Study Outcomes:**

This protocol will specify the primary outcome for efficacy, specified prior to knowledge of the results from the third International Stroke Trial, as the proportion of patients having a ‘favorable’ stroke outcome, defined by modified Rankin Score 0–1 at final follow-up at three- to six-months. The primary analysis will be to estimate the extent to which the known benefit of alteplase on modified Rankin Score 0–1 diminishes with treatment delay, and the extent to which it is independently modified by age and stroke severity. Key secondary outcomes include effect of alteplase on death within 90 days; analyses of modified Rankin Score using ordinal, rather than dichotomous, methods; and effects of alteplase on symptomatic intracranial hemorrhage, fatal intracranial hemorrhage, symptomatic ischemic brain edema and early edema, effacement and/or midline shift.

**Discussion:**

This collaborative meta-analysis of individual participant data from all randomized trials of intravenous alteplase vs. control will demonstrate how the known benefits of alteplase on ischemic stroke outcome vary across different types of patient.

## Introduction and rationale

For the treatment of patients with ischemic stroke, the only approved medical treatments for use within the first few hours of onset are thrombolytic therapy with intravenous (iv) recombinant tissue plasminogen activator (rt-PA) and aspirin. In 2004, a collaborative analysis of pooled individual patient data from some of the trials of iv rt-PA assessed the effects of time on benefit from treatment 1, an analysis that was subsequently updated in 2010 to include the results of the ECASS-3 2 and EPITHET 3 trials. These analyses demonstrated reliably that thrombolysis with iv rt-PA is both effective and safe when administered to particular types of patient within 4·5 h of having an ischemic stroke, and that treatment benefit diminishes with increasing treatment delay. However, several uncertainties remain regarding the potential effects of rt-PA when administered in different circumstances, as well as in different subgroups of patients 4. In this third phase of the collaborative analysis of pooled individual patient data from the rt-PA trials, results from the third International Stroke Trial (IST-3) [and, if possible, the Thrombolysis in Elderly Stroke Patients in Italy (TESPI) trial 5] will be included to help address these and other outstanding questions.

This statistical analysis plan describes the analyses that were agreed by the Stroke Thrombolysis Treatment (STT) Collaborative Group prior to becoming unblinded to the results from IST-3. The main protocol can be downloaded from the study Web site (http://www.ctsu.ox.ac.uk/research/meta-trials/stt).

## Comparisons of baseline measures between trials

Prior to performing analyses of estimated treatment effects (see below), we propose that descriptive and exploratory analyses will be performed to identify and display differences in baseline characteristics between the types of patient enrolled in the trials. In particular, statistical comparisons of baseline means (using *t*-tests) and prevalences (using chi-squared tests) between patients enrolled in IST-3 and patients enrolled in earlier trials will be performed. The baseline characteristics of patients in IST-3 who might have been expected to have been already eligible for treatment with rt-PA under guidelines at the time (e.g. patients presenting within 3 and within 4·5 h of stroke onset, particularly if aged <80 years), will also be presented because it is of interest to examine if reasons for the ‘uncertainty’ of benefit that prompted enrolment in IST-3 are evident. The rationale for performing these initial descriptive analyses is because an understanding of how IST-3 patients differ from those in the earlier trials might aid the interpretation of any apparent between-trial treatment differences that may arise (see section ‘IST-3 compared with earlier trials’).

## Primary prespecified analyses

It has already been established that thrombolysis with iv alteplase (rt-PA) is both effective and safe when administered to particular types of patient within 4·5 h of stroke onset, and that treatment benefit diminishes with increasing treatment delay 1,2. Consequently, any estimate of the overall effect for all patients randomized to rt-PA within six-hours of stroke onset provided by the analyses described in this document should not necessarily be used to guide the future use of treatment (or to revisit efficacy in presently recommended subgroups) because of the possibility that such an estimate might be diluted substantially by the results from IST-3 (which, through use of the ‘uncertainty principle’ in its design, recruited substantial numbers of patients in whom the effect of treatment may be proportionally smaller than that observed in previous trials, or even nonexistent). Rather, the analyses described in this document seek to promote a better characterization of the extent to which rt-PA treatment effects *vary in different types of patient*, with a concomitant improvement in the identification of subgroups of patients in whom treatment may be particularly beneficial, nonexistent, or even harmful.

### Primary outcome

The primary outcome will be the proportion of patients having a ‘favorable’ stroke outcome defined by modified Rankin Score (mRS) 0–1 at final follow-up at three- to six-months. If three-month outcome is available for a specific study, this will be used as the primary outcome in analyses (regardless of whether it was used as the primary analysis in the original study report). If the three-month outcome is not available, the next available follow-up point will be used, as long as that point is no more than 190 days after randomization (i.e. one-week beyond the patient's scheduled six-month visit). In IST-3, there was no three-month follow-up, and follow-up at six-months was done by postal questionnaire. For many patients there was a delay of some days or weeks before the form was returned and received by the co-ordinating center. Therefore, following the prespecified approach taken in IST-3 6, all completed six-month questionnaires in IST-3 will be included in analyses irrespective of when they were received by the co-ordinating center, with missing data imputed from the seven-day assessment using an algorithm that was found to work well among patients who had both measurements. In other trials, a conservative algorithm for imputing missing outcome data based on measurements made earlier than 90 days will also be used [as done previously 2]. This will assign a modified Rankin score of 5 if vital status is unknown or measurements were not available after baseline. If measurements are available after baseline in survivors, the last known score will be carried forward; otherwise for survivors a modified Rankin score of 5 will be imputed.

### Intention-to-treat (ITT) analyses and missing data

Wherever possible, analyses will include all randomized patients, irrespective of whether they subsequently received the intended treatment (i.e. according to the ITT principle). Patients with missing mRS at final follow-up will have a value imputed as described above. Any patients who withdrew their consent will contribute information only up to the point of withdrawal; thereafter, their missing data will be imputed.

The frequency of missing baseline data for each trial will also be assessed. Depending on the extent of missing data, a range of statistical approaches will be used, including imputing missing values with mean values from other patients in that trial; using missing value indicators in analyses; multiple imputation 7; and complete case analyses. The range of approaches are needed because none of these methods are guaranteed to yield unbiased results for tests of interaction (see ‘Primary analysis’ below) should the data be ‘missing not at random’ 8. In the unlikely scenario that these analyses give qualitatively different results the reasons for the differences will be explored and reported in publications. Otherwise, primary focus will be based on the simplest method of imputing missing data with the mean value seen among other patients in that trial.

### Primary analysis – after what treatment delay is benefit lost or does harm begin, and do age or stroke severity modify the proportional effect of rt-PA on stroke outcome?

A key feature of the main analysis is to estimate the extent to which the effect of allocation to rt-PA on a favorable stroke outcome (i.e. mRS 0–1) depends on particular patient characteristics recorded at randomization (i.e. treatment effect ‘modification’). To limit the potential for spurious results to arise from examination of multiple potential effect modifiers, the main analyses will be limited to three clinical characteristics that are anticipated to be particularly important: the time from stroke onset to treatment (hereon referred to as treatment delay), patient age, and stroke severity:

- *Treatment delay*. Treatment delay is defined as the time from symptom onset to treatment delivery. [In the IST-3 trial, for patients recruited in the open phase of the study who were allocated control, it is not possible to specify a time interval from onset to ‘treatment’ that is comparable to the time from onset to delivery of the rt-PA bolus dose. Following the approach outlined in the prespecified statistical analysis plan (SAP) for that trial, the delay from randomization to delivery of the bolus among patients allocated placebo will therefore be set to 18 min (the mean delay observed in patients randomized to rt-PA in IST-3).] It has previously been shown that there is a decay in treatment benefit with increasing treatment delay and that the declining benefit may even translate to increased mortality if treatment is delayed beyond 4·5 h 1,2. However, confidence intervals around the time at which benefit is lost and/or mortality is first encountered are wide. Presently, there is no evidence to suggest that treatment initiation beyond 4·5 h confers any net benefit and, as a consequence, such patients are not routinely offered treatment in current clinical practice. The addition of IST-3 data to the existing subgroup of patients treated between 4·5 and 6 h therefore carries the lowest risk of effect dilution when combining IST-3 with the existing collaboration (since selection is less affected by existing treatment recommendations) and so this subgroup will be specifically examined.

Regression analyses [see ‘Using regression to test for effect modification (two-way interactions)’] will be performed to estimate the relationship between treatment delay (handled as a continuous variable) and the log-odds ratio for the effect of allocation to rt-PA on a favorable stroke outcome, after adjustment for any other modifying effects on treatment of age and stroke severity (see b and c below). Specifically, analyses will test for linearity in the log-odds ratio (i.e. ‘log-linearity’ in the odds ratio) with increasing treatment delay, and will estimate the size of such an effect. Assuming such a trend is observed, further analyses will estimate the time at which the estimated benefit crosses zero, as well as the time at which the 95% confidence interval for the odds ratio first crosses one. (See ‘Key secondary analyses’ for a description of analyses of mortality.)

In addition to considering treatment delay as a continuous variable, the effect of allocation to rt-PA on stroke outcome in three subcategories of treatment delay will also be estimated: ≤3 h, >3 to ≤4·5 h; and >4·5 h. These estimates will also be adjusted for any modifying effects on treatment of age and stroke severity.

(b) *Patient age*. It is hypothesized that, for a given treatment delay and stroke severity, the proportional benefits of rt-PA on a favorable stroke outcome do not reduce with increasing patient age. It is important to test this hypothesis because the marketing authorization for rt-PA in some European countries is currently restricted to patients aged 80 or less (due primarily to limited direct randomized evidence in older patients), whereas nonrandomized controlled data and clinical guidelines support the use of rt-PA in the elderly 9–12. Thus, a key question to answer is whether the current European marketing authorization restriction, based on an upper age limit of 80, is justified.

Regression analyses will therefore be performed to test whether the proportional effect of allocation to rt-PA on a favorable stroke outcome varies (in a log-linear manner) with age at randomization (handled as a continuous variable), once adjustment is made for any treatment modifying effects of treatment delay and stroke severity. The effect of allocation to rt-PA on stroke outcome in patients ≤80 and >80 years of age will also be estimated (again, adjusted for other baseline covariates and treatment modifying effects of those covariates) and, if evidence of independent effect modification by age is observed, a test for difference in the two log-odds ratios will be performed (by comparing the difference in log odds ratios divided by its standard error against a standard normal distribution, a test that will be deemed nominally significant if the two-sided *P*-value is less than 0·05).

(c) *Stroke severity*. Stroke severity is defined by the National Institutes of Health Stroke Scale (NIHSS) score determined before treatment. The Marketing Authorization for rt-PA in stroke cautions against use in severe and mild stroke [Bibr b13]. Regression analyses will therefore be performed to test whether the proportional effect of allocation to rt-PA on a favorable stroke outcome varies across the NIHSS scores. As indicated above, it is anticipated that a ‘U-shaped’ relationship might exist between the proportional effect of treatment allocation and NIHSS score, with a smaller proportional effect of treatment being seen among patients with the lowest and highest scores 14. To test for such a relationship, regression analyses will be performed that allow the estimated treatment effect to vary with NIHSS score in such a manner (e.g. by inclusion of a quadratic interaction term in a model that additionally adjusts for any potential treatment modifying effects of treatment delay and patient age).

If evidence of effect modification is determined from this analysis, five subcategories of severity (NIHSS score: 0–4, 5–10, 11–15, 16–21, and ≥22) will be defined, and further analyses will be done to estimate the proportional treatment effect in each of these groups (after adjustment for the other baseline variables of age and treatment delay).

### Using regression to test for effect modification (two-way interactions)

As already stated, a key aspect of the main analysis is to estimate the extent to which treatment delay, age, and/or stroke severity modify the proportional effect of allocation to rt-PA on a favorable stroke outcome (i.e. mRS 0–1). Since these three baseline characteristics may be correlated with each other, if any one of them importantly modifies the proportional effect of treatment, then simple analyses of each in turn (e.g. through standard forest plots) might make it *appear* that the others do also even if the truth is that they do not (i.e. an apparent modifying effect on treatment may be *artificially induced* by a correlation existing with a ‘true’ effect modifier). Therefore, the main analyses will be done by fitting a logistic regression model (stratified by trial) with *simultaneous* adjustment for treatment allocation, the three key baseline characteristics (handled as appropriate for the particular hypothesis being tested; see above) and appropriate ‘two-way’ interaction terms with treatment allocation (i.e. treatment-by-delay, treatment-by-age, and treatment-by-severity). When handled as continuous variables, treatment delay, age, and stroke severity will be standardized prior to inclusion into any regression model (which will facilitate the interpretation of the ‘main’ effects in the presence of interaction terms but will not affect the statistical significance of any interaction terms). The statistical testing and estimation of the interaction terms will allow the following, hypothesis-driven, questions to be answered:

- To what extent does treatment delay modify the proportional effect of rt-PA on stroke outcome (taking into account any other independent relationships between age/stroke severity and treatment effect)?
- Does patient age modify the effect of rt-PA on stroke outcome (taking into account the independent relationships between treatment delay/stroke severity and treatment effect)?
- Does stroke severity modify the effect of rt-PA on stroke outcome (taking into account the independent relationships between treatment delay/age and treatment effect)?

As previously stated, the primary interest is not in the ‘main effect’ of treatment estimated across all the trials but rather the extent to which the treatment effect varies according to these three baseline characteristics. The most powerful test and reliable estimation of such effect modification is provided by an analysis that includes all IST-3 patients (irrespective of the overall results from IST-3). The results of these analyses for particular subgroups of patient defined by categories of treatment delay, age, and stroke severity will be shown graphically in forest plots that take their subgroup-specific relative risk estimates of the effect of allocation to rt-PA directly from the (relevant combination of) estimated regression coefficients from the appropriate regression models. Again, this is done most reliably by inclusion of all IST-3 patients into regression models that include the relevant interaction terms (which allow treatment effects to be estimated separately in each predefined subgroup).

Two-way interaction tests will be regarded as nominally significant if the two-sided *P*-value is less than 0·05, before adjustment for multiplicity (see ‘Interpretation of *P*-values for interaction’).

### Modification of effect modification (three-way interactions)

On the assumption that the analyses described above do suggest that the proportional effect on stroke outcome of allocation to rt-PA is modified by some baseline feature, then a natural subsidiary clinical question would be whether this effect modification itself might vary depending on one of the other baseline features. In particular, if, as expected from the results of the earlier trials, treatment delay is found to importantly modify the proportional treatment effect (independently of age and stroke severity), then two natural subsidiary questions would be

1. Does age have any impact on the extent to which treatment delay modifies the effect of treatment? In particular, it may be hypothesized that older age would shorten the time window during which rt-PA may safely be given.
2. Does stroke severity have any impact on the extent to which treatment delay modifies the effect of treatment? In particular, it may be hypothesized that it would be less safe to treat very severe strokes late because increasing depth and duration of ischemia may increase subsequent risk of reperfusion injury.

These questions can be answered using similar regression models to those described previously, but with the additional inclusion of appropriate ‘three-way’ interaction terms, one between age, treatment delay, and treatment allocation, and the other between stroke severity, treatment delay, and treatment allocation. (Note: the only other three-way interaction term that could be fitted, between age, stroke severity, and treatment allocation, would be included if either the interaction between age and treatment or the interaction between stroke severity and treatment were found to be independently significant.)

Three-way interaction tests will be regarded as nominally significant if the two-sided *P*-value is less than 0·05, before adjustment for multiplicity (see ‘Interpretation of *P*-values for interaction’).

### Interpretation of *P*-values for interaction

Statistical tests for effect-modification (and modification of effect modification) will be provided by the *P*-values corresponding to the relevant two- or three-way interactions described above. In general, a two-sided *P*-value <0·05 will be considered as evidence supporting true effect modification. The rationale for using a *P*-value of 0·05, rather than 0·1 for instance (which would increase the power of the test), is that the probability of incorrectly claiming evidence for effect modification increases both with the nominal significance level and the number of tests being performed. For example, for any single regression model which includes three 2-way interaction terms, the probability of one or more ‘false-positive’ results is increased from about one in seven when using the 5% significance level to one in four when using the 10% significance level. Such a false-positive finding could be seriously damaging to clinical practice if it meant that inappropriate regulatory changes were made to the treatment indication as a result. Nonetheless, even with a *P*-value for interactions of 0·05, the probability of a false-positive result arising is not negligible. Thus, *P*-values will always be interpreted based on their actual value, rather than merely whether or not they are above or below a necessarily arbitrary value.

### IST-3 compared with earlier trials

One reason why the main analyses have been specified to allow assessment of how the effect of rt-PA varies depending on treatment delay, age, and stroke severity is because patients in whom rt-PA might be anticipated to have a reduced, or no, benefit (based on these criteria) are likely to be overrepresented in the IST-3 trial. Therefore, in a further regression model, an additional two-way interaction term between randomization into the IST-3 trial (a binary indicator) and treatment allocation will be fitted (in a model that also includes IST-3 as a main effect) to test whether or not, after allowing for the effect of treatment to vary according to treatment delay, patient age, and stroke severity, there remain any significant differences between the result of IST-3 and the pooled result from the other trials (i.e. are there unexplained differences). Specifically, the difference in minus twice the log-likelihood statistic between two nested models, one including an interaction term between enrollment in IST-3 and treatment with rt-PA and one not including such an interaction, will be tested against a chi-squared distribution with 1 degree of freedom. (Note: To allow estimation of main and interaction effects involving comparisons of IST-3 patients with patients recruited into other trials, this regression model will need to be unstratified, effectively resulting in the pooling of patients from the other trials into a single group.)

## Secondary analyses

### Key secondary analyses

- Effect of treatment allocation on death within 90 days, analyzed using Cox proportional hazards regression, stratified by trial, with failure time set to time from randomization to death/censoring time. The potential for effect modification will be assessed by the addition of interaction terms to the model.
- An analysis of the effect of treatment allocation on mRS using a ‘sliding dichotomy approach’ (also sometimes referred to as ‘responder analysis’, ‘prognosis-adjusted analysis’, or ‘patient-specific analysis’) 15. For this approach, a favorable stroke outcome is defined individually for each patient based on their risk profile at randomization (rather than applying one rule for all patients).

### Other secondary outcomes

Further analyses will be done to assess the effect of allocation to rt-PA on

- Symptomatic intracranial hemorrhage, defined using PH2 or PH2 with the SITS-MOST criterion of a deterioration of ≥4 NIHSS points.
- Fatal intracranial hemorrhage (PH2 and death within seven-days).
- Symptomatic ischemic brain edema (brain tissue swelling associated with neurological deterioration by ≥4 NIHSS points).
- Early edema, effacement and/or midline shift.

Time to event outcomes will be analyzed using Cox proportional hazards regression, stratified by trial, with failure time set to time from randomization to outcome. Where there are a sufficient number of events (at least 10 per predictor variable), the potential for effect modification will be assessed by the addition of interaction terms to the model.

### Other analyses of mRS

A number of other preplanned secondary analyses of stroke outcome will be conducted:

- An analysis using an identical analytic approach of the dichotomous mRS outcome, but rather than modeling the probability of the outcome mRS 0–1, using instead the outcome mRS 0–2 (then, additionally, each of the outcomes mRS 0, mRS 0–3, mRS 0–4, and mRS 0–5).
- An analysis of the distribution of mRS using an approach proposed by Howard *et al*. 16 (Briefly, the approach is akin to the Mann–Whitney *U*-test in that it focuses on whether a randomly chosen actively treated patient is more or less likely to have a better outcome than a randomly chosen placebo-treated patient. This analysis will also be stratified by study and assess the potential for effect modification by the three prespecified baseline characteristics.)
- Analyses of mRS across the whole spectrum using the Cochran–Mantel–Haenszel test followed by the proportional odds model.

### Modification of effects of rt-PA on the primary outcome by other baseline characteristics

In addition to the three primary potential effect modifiers (treatment delay, age, and stroke severity), under a secondary set of hypotheses, further analysis of potential effect-modification will be performed for other baseline characteristics. Prioritized among these are blood pressure, blood glucose, and body temperature on admission. At a later stage other variables will also be examined, including sex, presence of atrial fibrillation on prerandomization electrocardiogram, baseline imaging features (e.g. presence of hyperdense middle cerebral artery, visible early ischemic tissue changes, ischemic leukoencephalopathy), stroke clinical syndrome, side of lesion (left vs. right hemisphere), concomitant treatments at baseline (e.g. antiplatelets, oral anticoagulants, statins), predicted prognosis, predicted risk of symptomatic intracranial hemorrhage, and tPA dose.

Any apparently significant interactions arising from these analyses will be interpreted with appropriate caution (depending on the extent of their statistical significance), and, in general, may be considered as ‘hypothesis generating’ only.

### Other prespecified analyses

In an additional analysis, the impact of affected hemisphere (left vs. right) on the log-odds ratio for the effect of allocation to rt-PA on a favorable stroke outcome in people with a low NIHSS score (≤7) will be assessed. In particular, it is hypothesized that among people with a low NIHSS score, treatment with rt-PA will have little or no benefit among patients with stroke in the right hemisphere but substantial benefit among patients with stroke in the left hemisphere.

#### Collaborating trials

ATLANTIS (Gregory Albers, James Grotta, Maarten Lansberg, Jean Marc Olivot); ECASS-1, ECASS-2, ECASS-3 (Erich Bluhmki, Werner Hacke, Markku Kaste, Kennedy Lees, Ruediger von Kummer, Nils Wahlgren, Gregory J del Zoppo); EPITHET (Stephen Davis, Geoffrey Donnan, Mark Parsons); IST-3 (Peter Sandercock, Joanna Wardlaw, Richard Lindley, Gordon Murray, Geoff Cohen, William Whiteley); NINDS (James Grotta, Patrick Lyden, John Marler, Barbara Tilley); TESPI (Danilo Toni).

#### STT Statistical Analysis Center and Secretariat

Colin Baigent, Kate Bird, Lisa Blackwell, Erich Bluhmki, Jonathan Emberson, Heather Halls, Lisa Holland, George Howard.
